# A1 adenosine receptor antagonist induces cell apoptosis in KYSE-30 and YM-1 esophageal cancer cell lines

**DOI:** 10.37796/2211-8039.1394

**Published:** 2023-03-01

**Authors:** Parisa Zeynali, Marie Saghaeian Jazi, Jahanbakhsh Asadi, Seyyed Mehdi Jafari

**Affiliations:** a Metabolic Disorders Research Center, Golestan University of Medical Sciences, Gorgan, Iran; b Department of Biochemistry and Biophysics, Faculty of Medicine, Golestan University of Medical Sciences, Gorgan, Iran

**Keywords:** Esophageal cancer, A1 adenosine receptor, DPCPX, Apoptosis

## Abstract

**Background and aim:**

Adenosine A1 receptor (AA1R) has been shown to have an inhibitory effect on cell growth in several cancers; however, its function in esophageal cancer is still unclear. In this study, we examined the effect of AA1R on cell growth and apoptosis in esophageal cancer cells.

**Materials and methods:**

In this study, YM-1 and KYSE-30 esophageal cancer cell lines were cultured. AA1R gene expression was determined by quantitative Real-time Polymerase Chain Reaction (qRT-PCR). As well, the AA1R antagonist (DPCPX) effect on cell viability was evaluated by the MTT assay. Moreover, apoptosis was assessed by annexin-V and propidium iodide staining, and the caspase-3/7 activity assay kit.

**Result:**

qRT-PCR results indicated that the AA1R was expressed in YM-1 and KYSE-30 cells. In addition, DPCPX significantly decreased cell proliferation in both cell lines. Furthermore, the A1AR antagonist induced apoptosis in KYSE-30 and YM-1 cells. After treatment of both cell lines with DPCPX, the caspase 3/7 activity was increased.

**Conclusion:**

Our finding indicates the AA1R antagonist induces apoptosis through caspase 3/7 activation and can be considered a potential target in esophageal cancer therapy.

## 1. Introduction

Esophageal cancer (EC) is the eighth most prevalent type of cancer and the sixth leading cause of cancer death worldwide [1]. The high mortality rate of EC is a consequence of inadequate treatment, chemo-resistance, and metastasis in patients with advanced cancer [2]. Recent studies on the molecular characteristics of cancer cells have led to the identification of novel molecular targets in cancer therapy. In this regard, inhibition of the oncogenic intracellular signaling pathways may reduce the proliferation of cancer cells [3].

The tumor microenvironment (TME) contains various components. Multiple immunological and metabolic changes are triggered by tumor hypoxia, which controls tumor growth and neutralizes anti-tumor immune responses [4]. The adenosinergic system embodies immune response control mechanisms. Adenosine builds up at the tumor site when oxygen is not present [5,6].

Adenosine, an ATP-derived nucleoside, stimulates apoptosis in cancer cells through various signal transduction pathways by activating four adenosine receptors; A1, A2a, A2b, and A3 receptors [7,8]. Extrinsic and intrinsic are the two main pathways of apoptosis. In the intrinsic apoptosis pathway, the conjunction between cytochrome *c* released into the cytosol from perturbed mitochondrial with apoptotic protease-activating factor 1 and inactive caspase 9 leads to the formation of the apoptosome complex. Then, adenosine triphosphate hydrolyzes by complex, and leads to caspase-9 activation. After all, the initiator caspase-9 cleaves and executioner caspase3/6/7 actives. The extrinsic pathway that triggers apoptosis is initiated by a death receptor such as Fas receptors, tumor necrosis factor (TNF) receptors, and TNF-related apoptosis-inducing ligand (TRAIL). These receptors activate caspases and cause apoptosis through the extrinsic pathway when stimulated by ligands that are related to them [9].

According to previous studies, the adenosine A1 receptor (AA1R) plays a critical role in the pathogenesis of different cancers, such as breast [10], colorectal [11], melanoma [12], and renal cancers [13]. Hosseinzadeh et al. discovered that AA1R agonists like CHA or R-PIA, can inhibit cell proliferation in the liver and renal adenocarcinoma cancer cell lines, HepG2 and CACO2, respectively [14]. These results indicate the possible role of AA1R in regulating cancer cell proliferation and illustrate its potential for cancer targeting. Although earlier research demonstrated the role of AA1R in various cancer cell lines, its role in esophageal cancer has not been clarified. Therefore, the present study aimed to investigate the effect of AA1R on proliferation and apoptosis of esophageal cancer cells.

## 2. Material & methods

### 2.1. Cell culture

The Ym-1 cell line was obtained from epithelial cells of human esophageal squamous cell carcinoma as described previously [15]. YM-1 is an epithelial cell line derived from ESCC tumor tissue of 59 years old Iranian female patient with a doubling time of about 40 h, and well defined STR profile [15] (https://www.cellosaurus.org/CVCL_AP55). KYSE-30 cell line (human esophageal squamous cell carcinoma) was obtained from Pasteur Institute (Tehran, Iran). The KYSE-30 is an ESCC cell line derived from a 64 years old male Asian patient with a doubling time of about 20 h harboring mutations in p53 (IVS6-2A > G) and HRAS (c.182A > T) (https://www.cellosaurus.org/CVCL_1351). Cells were cultured in the Dulbecco’s Modified Eagle Medium (DMEM/F12, Gibco) supplemented with 10% fetal bovine serum (Gibco, Germany), 100 U/mL penicillin (BIO IDEA, IRAN), and 100 μg/mL of streptomycin (Bioidea, Iran), at 37°C in a humid environment with 5% CO_2_. In 80% confluency, cells were detached by Trypsin/EDTA (BIO IDEA, IRAN). Then, cells were seeded in 6-well for gene expression, apoptosis, and caspase assay. As well, cells were seeded in 96-well plates for MTT assay. After 24 h, cells were treated with appropriate concentrations of the AA1R agonist CPA or/and antagonist DPCPX (Sigma, USA) for 48 h. The dimethyl sulfoxide (DMSO) was ordered from Sigma (Sigma, USA).

### 2.2. Real-time polymerase chain reaction

Total RNA was extracted using the RNX plus reagent (Sinaclon, Iran) following the manufacturer’s instructions. The RevertAid First Strand cDNA Synthesis Kit (Yekta Tajiz, Iran) was used to reverse-transcribe 1000 ng of total RNA after DNase I treatment into cDNA by the manufacturer’s instructions. The Maxima SYBR Green (Yekta Tajhiz, Iran) was used for quantitative Real-time PCR (qRT-PCR) via specific primers. Primer sequences are listed in.

Table 1. Gene expression was measured by Step One Plus, a Real-time PCR instrument (Applied Biosystems, USA). The real-time PCR program was designed as follows: 1) denaturation step. 10 min at 95^°C^, 15 s; 2, and 3) annealing and extension step. 1 min at 60^°C^. The relative gene expression level of AA1R between YM-1 and KYSE30 cancer cells was calculated by the 2^−^^ΔΔ^^Ct^ formula using GAPDH as a reference gene. The melting temperature (TM) of particular amplification products (supplementary file 1) were determined using the melt curve analysis (0.3°C increments from 60 to 95°C).

### 2.3. MTT assay

The 96-well plate was filled with 1 × 10^4^ cells per well. Drugs were dissolved in a complete DMEM medium and cells were treated with those concentrations. Cell viability was assessed after 48 h, using an MTT assay. Briefly, cells were incubated with 20 μl MTT tetrazolium salt at 37^°C^ for 3 h. Finally, the formazan crystals were dissolved with DMSO (100 μl/well), and a plate reader was used to determine the optical density (OD) at wavelength 570 nm. By comparing the optical density of each sample with the optical density of the control group, the percentage of cells was calculated.

### 2.4. Apoptosis assay

The flow cytometry assay was performed to calculate the percentage of apoptotic cells after annexin V and PI labeling. The 4 × 10^5^ cells per mL were examined for each sample. After 48 h treatment with different concentrations of DPCPX (10 μM and 100 μM), cells were detached and rinsed by phosphate buffer saline (PBS) and resuspended in binding buffer. After adding annexin-V-FITC to cell suspensions, the test was performed by the FACSCalibur flow cytometer.

### 2.5. Caspase-3/7 activity

Using colorimetric assay kits by the manufacturer’s protocol, caspase-3/7 activity was assessed (Kiazist, Iran). Specifically, cells were treated with DPCPX for 48 h, rinsed with PBS, lysed by lysis buffer, and centrifuged for 10 min at 10,000 rpm. Then, 50 μl of reaction buffer, 5 μl of substrates specific for caspase-3/7 (DEVD), and cell lysate were combined. The mixture was incubated at 37^°C^ for 1 h. Finally, absorbance was measured at 405 nm with a microplate reader (BioRad, Hercules, CA, USA).

### 2.6. Statistical analysis

To determine the statistical significance between groups, version 20 of SPSS software was used to perform the Shapiro–Wilk test (to assess normality). The One-Way Analysis of Variance (ANOVA) followed by the Tukey post-hoc test. *P* < 0.05 was used to determine statistical significance. The mean and standard deviation (SD) was used to present the quantitative data.

## 3. Results

### 3.1. Expression of AA1R

**Fig. 1:**
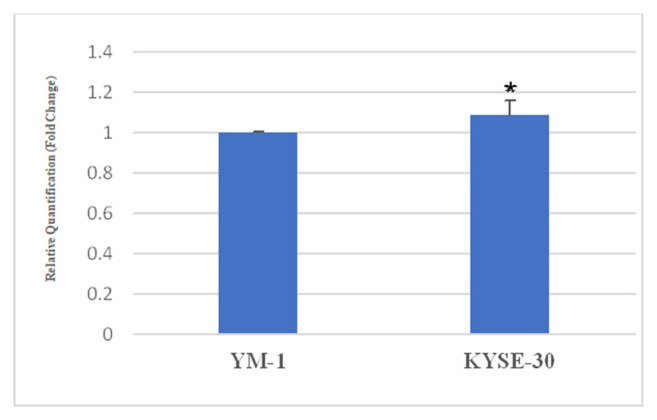
The gene expression of A1AR. The expression of AA1R in YM-1 and KYSE30 cells was evaluated by qRT-PCR. The expression of AA1R was more in KYSE30 cells compared to YM-1 cells. *P < 0.05 compared to controls.

To demonstrate the AA1R expression in esophageal squamous YM-1 and KYSE-30 cell lines, we used qRT-PCR. As indicated in Fig. 1, AA1R was expressed in both cell lines; however, the expression of AA1R was higher in the KYSE-30 cells compared to YM-1 cells *(P* < 0.05).

### 3.2. Effect of the AA1R antagonist on cell viability

**Fig. 2:**
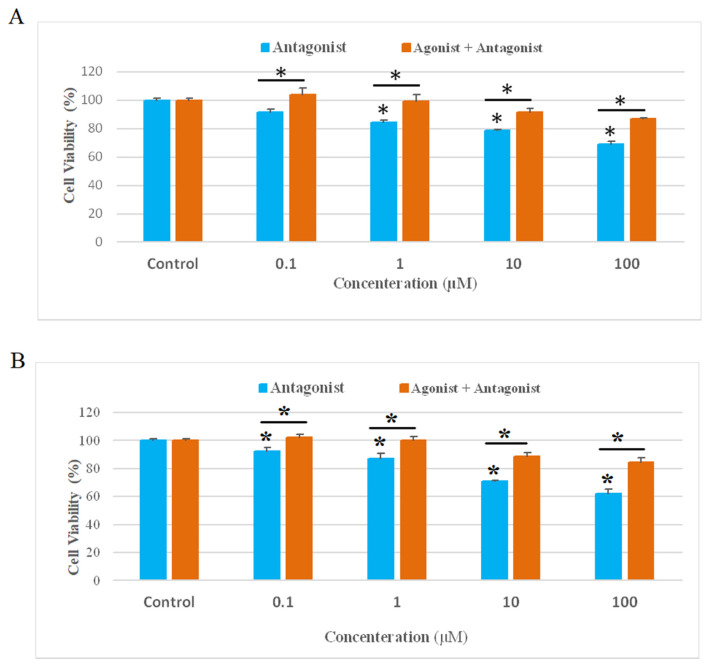
The effects of AA1R antagonist on cell viability. YM-1(A) and KYSE-30 (B) cancer cells were treated with various concentrations of antagonist for 48 h in the presence and absence of agonist, and cell viability was evaluated with MTT assay. *P < 0.05 compared to controls.

The inhibitory effect of the DPCPX on the proliferation of the YM-1 and KYSE30 cell lines was investigated using the MTT assay. Finally, the cell viability percentage was evaluated in different concentrations (0.1, 1, 10,100 μM) of DPCPX and incubated at 37^°C^ for 48 h. Increasing the concentration of DPCPX in both cancer cell lines showed a significant decrease in living cells compared to the control group, and this effect was concentration-dependent (P ≤ 0.05). To confirm the effect of DPCPX on AA1R, cells were pretreated with 1 μMof CCPA as the agonist AA1R for 30 min and then were treated with different concentrations of DPCPX (0.1–100 μM). Consequently, we found that the CCPA, as an AA1R agonist, can reverse the DPCPX-induced cytotoxicity effect on YM-1 and KYSE30 cell lines (Fig. 2A and B).

### 3.3. The effect of AA1R antagonist on apoptosis

**Fig. 3:**
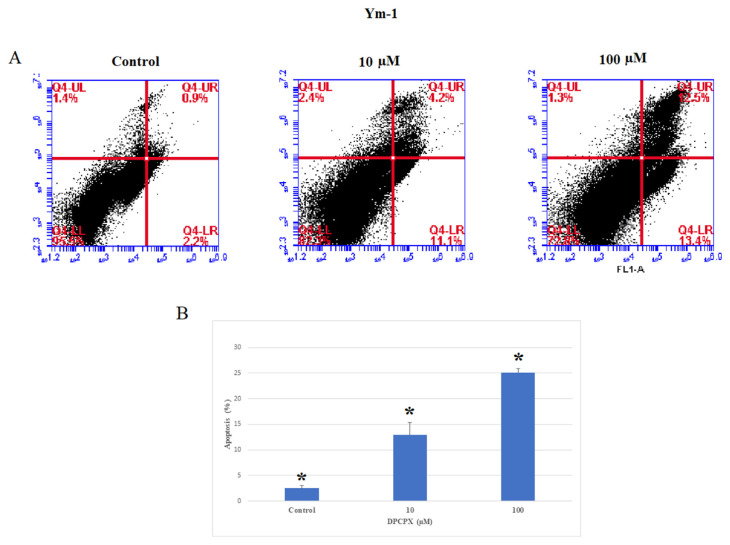
The effects of the AA1R antagonist on cell Apoptosis in YM-1 cancer cells. A. YM-1 cancer cells were treated with concentrations of 10 and 100 μM the antagonist for 48 h, and cell apoptosis was evaluated with Annexin-PI staining by flow cytometry. B. After treatment with an antagonist, the percentage of apoptosis significantly increased. *P < 0.05 compared to controls.

**Fig. 4:**
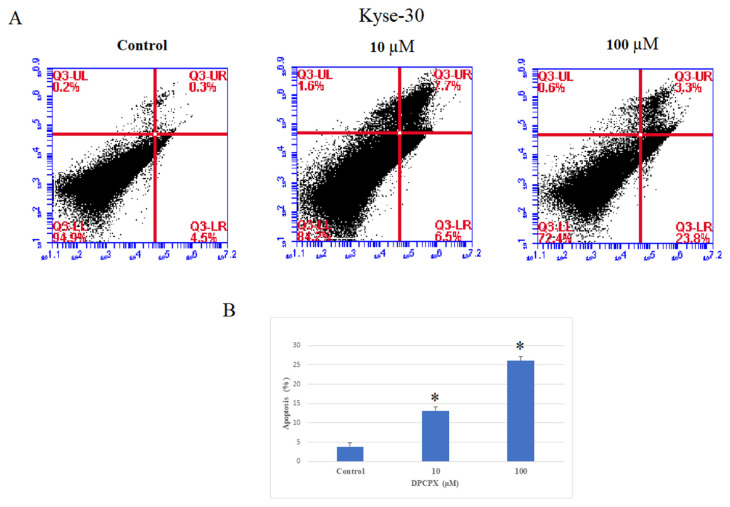
The effects of the AA1R on cell Apoptosis in KYSE30 cancer cells. A. KYSE-30 cancer cells were treated with concentrations of 10 and 100 μM antagonist for 48 h, and cell apoptosis was evaluated with Annexin-PI staining by flow cytometry. B. After treatment with an antagonist, the percentage of apoptosis significantly increased. *P < 0.05 compared to controls.

To determine the apoptotic potential of DPCPX, KYSE-30 and YM-1 cells were treated with the 10 and 100 μM concentration of DPCPX for 48 h (Figs. 3 and 4), and then the proportion of apoptotic cells was determined by flow cytometry. Our findings illustrated the percentage of apoptosis significantly increased in YM-1 and KYSE-30 cell lines (*P* < 0.05). The total apoptosis percentage of YM-1 and KYSE-30 cell lines at 100 μM concentration of DPCPX were 25.9% and 27.1%, respectively.

### 3.4. The effect of AA1R antagonist on caspase 3/7

**Fig. 5:**
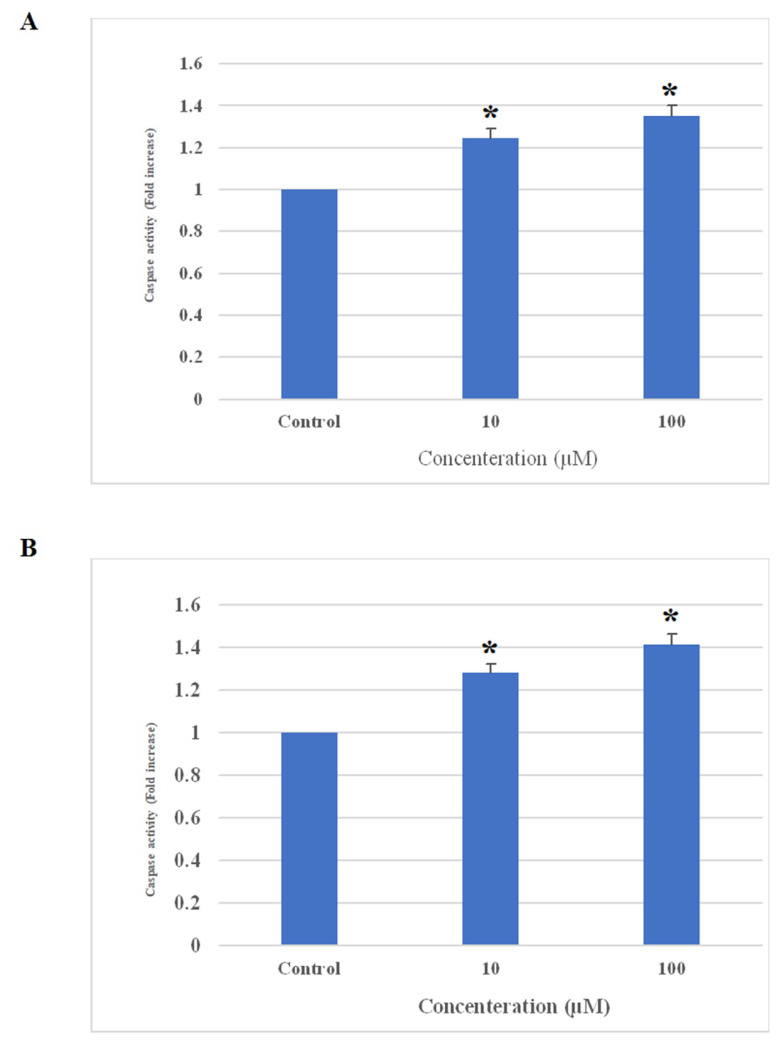
The effects of AA1R on Caspase 3/7. A (YM-1) and B (KYSE-30) cancer cells were treated with the concentrations of 10 and 100 μM the antagonist for 48 h, and absorbance was measured at 405 nm. A significant increase in caspase 3/7 activity was indicated after treatment with an AA1R antagonist. *P < 0.05 compared to controls.

We measured caspase-3/7 activation as a key biomarker of apoptosis induction. Our data indicated (Fig. 5) a significant increase in caspase 3/7 activity after treatment with DPCPX after 48 h *(P* < 0.05).

## 4. Discussion

Adenosine is a purine nucleoside with several physiological activities, including prevention of platelet aggregation, stimulation of mast cells, and influences on the cardiovascular system, nervous system, and immune system [16]. Adenosine has recently been essential for many physiological processes, including cell proliferation [17]. Adenosine and its analogs have also been shown to cause apoptosis in various cell types, including astrocytoma and endothelial cells. In vitro and in vivo studies have shown that adenosine has anti-proliferative properties [18]. In particular, adenosine was investigated in some cancer cell lines as a potential anti-tumor agent [19]. Regarding the various functions of the adenosine A1 receptor in certain diseases, it has been proposed that this receptor may act as a potent regulator of normal and tumor cell growth by having both pro and anti-apoptotic effects [20]. The overexpression of AA1R is indicated in various cancer cell lines such as breast [21], ovarian [22], Renal [13], and glioma [23]. First, we looked into the expression of adenosine A1 receptors in KYSE-30 and YM-1 esophageal cancer cells. Our findings demonstrated that KYSE-30 had a higher expression of AA1R than YM-1. The finding of this study supports the inhibitory effect of AA1R antagonist on YM-1 and KYSE-30 esophageal cancer cell line proliferation, despite conflicting data on the role of AA1R in various cancers. Consistent with our results, Zhou et al. showed the AA1R antagonist inhibits cell growth in renal cell carcinoma [13]. As well, Dastjerdi et al., in a study on the AA1R, showed the antagonist of this receptor inhibits cell viability in the breast cancer cell [10]. In contrast, Daniel et al. indicated the AA1R agonist enhances the rate of cell proliferation in glioma tumor cells [23]. Another study also showed the AA1R agonist inhibited proliferation in gastric and colon cancer cell lines [24,25].

Apoptosis is an essential factor for determining the cell growth rate [26]. To clarify the DPCPXinduced cell death, we performed the apoptosis assay. According to our research, DPCPX causes apoptosis. Moreover, because various upstream pathways for final apoptotic execution depend on caspase-3/7 induction, we evaluated the effect of AA1R antagonist on the activity of caspase 3/7.

We showed that DPCPX induced caspase 3/7 activity. In a study by Zhou et al., they found the AA1R antagonist induced apoptosis in kidney cancer cells [13]. In another study by Choi et al., they were shown that adenosine affects the AA1R and subsequently induces apoptosis and activates caspases-3 in laryngeal cancer cells [27]. In contrast, the AA1R agonist induced apoptosis in colon cancer cells, and the AA1R antagonist inhibited apoptosis [24]. Another study revealed the induction of apoptosis in MCF-7 breast cancer cells through caspase-3 after treatment with an AA1R antagonist [19]. Recently, in a previous study, we indicated the AA1R antagonist induces apoptosis through caspase 3/7 in the A549 lung cancer cells [28].

## 5. Conclusion

According to the study, the AA1R antagonist induces apoptosis by activation of caspase 3/7 in the esophageal cancer cells, which makes it a potential therapeutic target for the disease.

## Figures and Tables

**Table 1:** Primer sequences for AA1R and GAPDH.

Number	Primer Name	Sequence	Length
1	AA1R Forward	CCACAGACCTACTTCCACACC	19
2	AA1R Reverse	TACCGGAGAGGGATCTTGACC	21
3	GAPDH Forward	AAGGTGAAGGTCGGAGTCAA	22
4	GAPDH Reverse	AATGAAGGGGTCATTGATGG	21

## Appendix

Fig. S1 Graph Charts of the Melt Curve for AA1R Genes Products.
