# Supplementary figures and images for: A1 adenosine receptor antagonist induces cell apoptosis in KYSE-30 and YM-1 esophageal cancer cell lines
Source: Biomedicine (Taipei). 2023 Mar 1;13(1):54–61. doi: 10.37796/2211-8039.1394 (PMC10166249; doi:10.37796/2211-8039.1394)

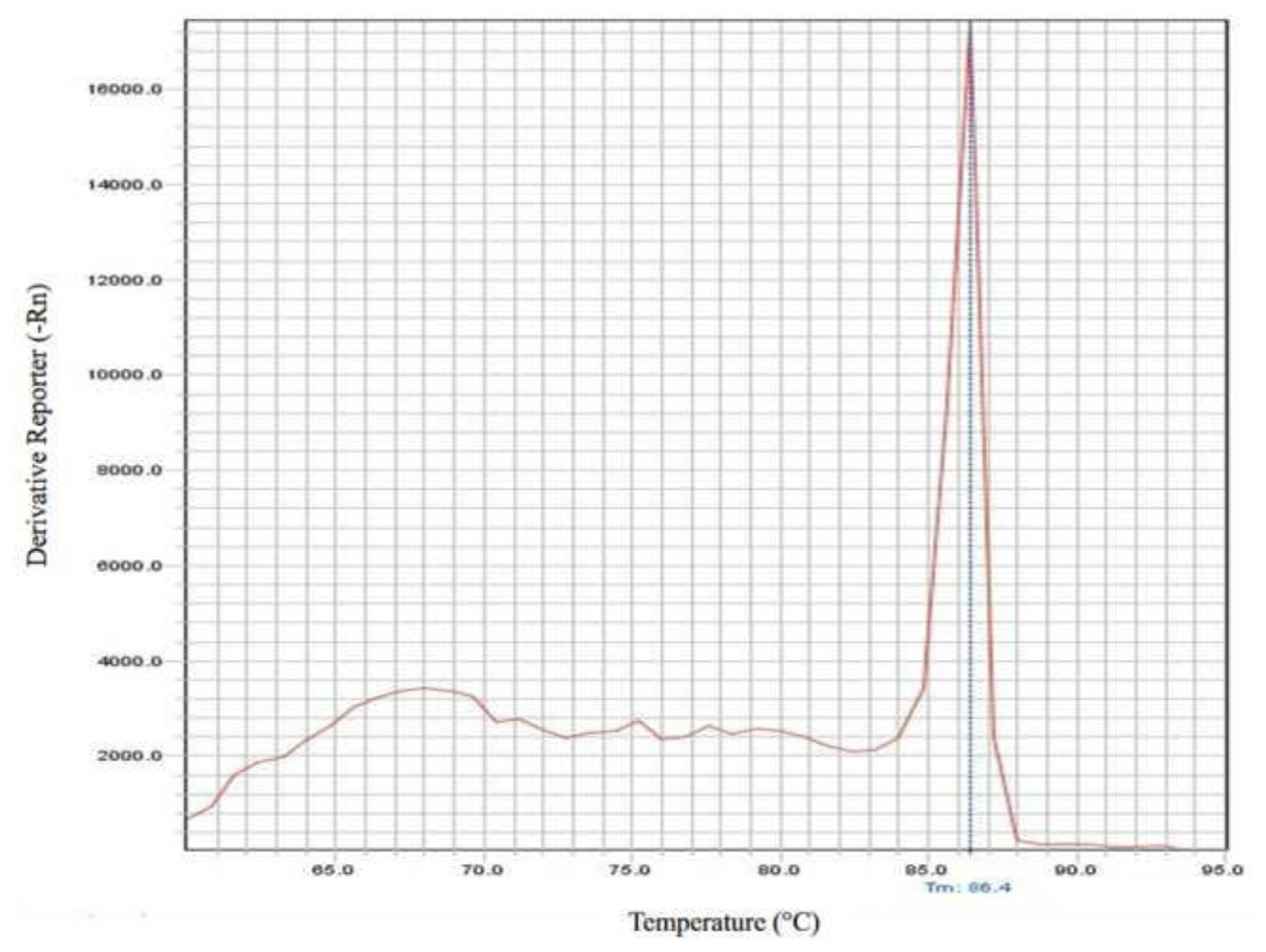

Supplement: Fig. S1 — Graph Charts of the Melt Curve for AA1R Genes Products. [file bmed-13-01-054s1.tif]
